# Microbiota-derived metabolites in inflammatory bowel disease

**DOI:** 10.1007/s00281-025-01046-9

**Published:** 2025-03-04

**Authors:** Martina A Guggeis, Danielle MM Harris, Lina Welz, Philip Rosenstiel, Konrad Aden

**Affiliations:** 1 Institute of Clinical Molecular Biology, Kiel University and University Medical Center Schleswig-Holstein, Rosalind Franklin Straße 11, Campus Kiel, 24105 Kiel, Germany; 2 Department of Internal Medicine I, Kiel University and University Medical Center Schleswig-Holstein, Rosalind Franklin Straße 11, Campus Kiel, 24105 Kiel, Germany; 3 Division Nutriinformatics, Institute for Human Nutrition and Food Science, Kiel University, Kiel, Germany

**Keywords:** Inflammatory bowel diseases, Microbiome, Metabolites, Treatment, Personalized medicine

## Abstract

Understanding the role of the gut microbiota in the pathogenesis of inflammatory bowel diseases (IBD) has been an area of intense research over the past decades. Patients with IBD exhibit alterations in their microbial composition compared to healthy controls. However, studies focusing solely on taxonomic analyses have struggled to deliver replicable findings across cohorts regarding which microbial species drive the distinct patterns in IBD. The focus of research has therefore shifted to studying the functionality of gut microbes, especially by investigating their effector molecules involved in the immunomodulatory functions of the microbiota, namely metabolites. Metabolic profiles are altered in IBD, and several metabolites have been shown to play a causative role in shaping immune functions in animal models. Therefore, understanding the complex communication between the microbiota, metabolites, and the host bears great potential to unlock new biomarkers for diagnosis, disease course and therapy response as well as novel therapeutic options in the treatment of IBD. In this review, we primarily focus on promising classes of metabolites which are thought to exert beneficial effects and are generally decreased in IBD. Though results from human trials are promising, they have not so far provided a large-scale break-through in IBD-therapy improvement. We therefore propose tailored personalized supplementation of microbiota and metabolites based on multi-omics analysis which accounts for the individual microbial and metabolic profiles in IBD patients rather than one-size-fits-all approaches.

## Introduction

Exploring the dynamic relationship between the human body and its microbiota in health and disease has been a subject of intense research in the recent decades. Especially in the gut, where the microbial density is at its highest in the human body and trillions of bacteria, fungi, viruses, archaea, and bacteriophages reside, the microbiota are crucial in shaping the functions and processes of the human body [1–4]. Besides supporting the gut in its digestive functions, the microbiota play a major role in shaping the body’s immune response. This has for example been shown in animal studies comparing germ-free (GF) and conventionally raised animals, where an altered immune maturation and response in germ-free animals was observed [5, 6]. A key mechanism through which the microbiota influence immune maturation is via the release of metabolites. These metabolites may directly alter immunometabolic processes or bind to specific receptors, thereby modulating innate and adaptive immune functions [7–9].

**Fig. 1:**
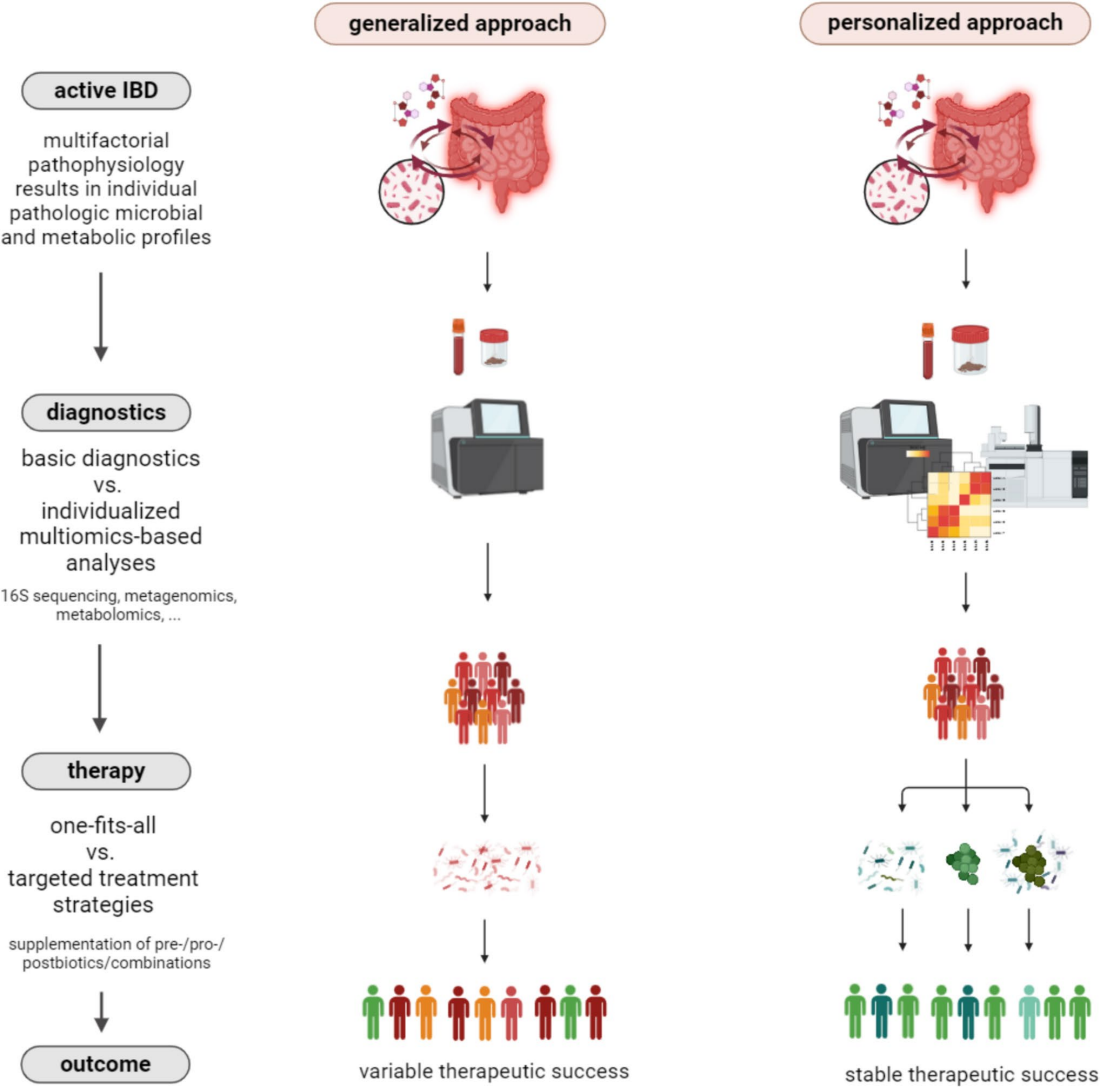
Tailored microbiome- and metabolite-based approaches as personalized therapeutic strategies in IBD. Visualization of a generalized approach using basic diagnostics and a common treatment concept administering the same pre-, pro- or post-biotic to everyone (left side of figure). Comparison to using a personalized approach with individualized multi-omics-based diagnostics followed by personalized supplementation based on the microbial taxa and metabolic profiles measured (right side of figure). (Created with BioRender. Aden, K. (2024) BioRender.com/q72k427)

It is hence not surprising that in human studies numerous associations have been found between chronic inflammatory diseases (CIDs) and altered microbial and metabolic signatures [10–13], and metabolic properties of the intestinal microbiota have been shown to causally modify inflammatory reactions in animal models of CIDs [14–16]. As an example, alterations in the microbiota are considered a hallmark of inflammatory bowel disease (IBD) including Crohn’s disease (CD) and ulcerative colitis (UC), which are chronic relapsing inflammatory diseases of the gut. The exact mechanism leading to chronic inflammation in IBD has not been fully elucidated due to the complex, multifactorial interactions between the host immune system, the microbiota and environmental stimuli [17]. Notably, the prevalence of these diseases has been increasing worldwide over the last decades [18] and a high proportion of patients still fail to respond to current therapeutic options in the long term [19]. Although it has not conclusively been shown whether they are a cause or consequence, numerous studies in animal models of IBD have implicated a mechanistic impact of microbial metabolites and/or functions in onset and progression of the disease [20, 21]. Apart from their potential as biomarker candidates, further research on the functional role of microbes in IBD, e.g., to unveil mechanisms influencing disease course and therapy response in IBD, is thus of great promise to provide novel therapeutic avenues in these debilitating diseases. Given the immediate proximity of the intestinal microbiota and their metabolites to the site of inflammation in IBD, understanding the language of interactions with the gut epithelium and immune system could help to identify such actionable therapeutic targets. The aim of this review is to discuss the relevance of microbial metabolism in IBD and its clinical implications for novel diagnostic and therapeutic approaches. Ultimately, we propose that personalized strategies will be key to clinical implementation (Fig. 1).

## The gut microbiota and metabolome

The development of novel omics-based profiling technologies, such as next generation sequencing (NGS) and metabolomics, has revolutionized our approach to describing microbial community composition and metabolic inventories in health and disease. Nevertheless, understanding the mechanisms of how the microbiota and host shape each other is far from being complete.

Human genetic factors can influence the microbial composition, as changes in host genes coding for antimicrobial functions of the gut can affect its colonization [22, 23]. In addition, countless environmental factors including diet and medication use, as well as intrinsic factors such as age and BMI influence the composition of the intestinal microbiota [24].

Conversely, the microbiota shape the human body by fulfilling a variety of physiological functions, including providing protection from pathogens through several mechanisms. For one, colonization with commensals leads to competition for biological niches and nutrients, limiting proliferation of other potentially harmful microorganisms. Furthermore, microorganisms shape the immune system of the host, which in turn modulates the composition of the commensals. This effect is mediated not only through direct binding of bacterial components to host receptors or antibodies, but also through the secretion of metabolites which can be absorbed in host cells and enter the systemic circulation [25]. Bacteria can produce metabolites through *de novo* synthesis, conversion of dietary components including medication, or metabolism of substances secreted by the host such as bile acids [26]. Therefore, the concentration of these metabolites can to a certain degree be influenced by the host itself, as for example a fiber-rich diet can increase the synthesis of short-chain fatty acids (SCFAs) [27]. Improvements in the measurement of metabolites through both targeted and untargeted approaches have led to a greater understanding of the human metabolome, although the differentiation between bacterial derived metabolites and human metabolites can be challenging. Recently, Neveu et al. created a database of metabolites by manually analyzing existing peer-reviewed articles, identifying 462 compounds fully or partially synthesized by the gut microbiota. The chemical classes the bacterial metabolites most frequently belonged to were amino acids and peptides, phenylpropanoids and polyketides, fatty acids, bile acids, and alcohols and derivatives [28].

Determining a “normal” microbiota with its accompanying metabolic profile is almost impossible given the wide variation across healthy individuals and within individuals at different timepoints. Correspondingly, it is demanding to assess which microbial species and metabolites are compromised in an individual patient, rendering a targeted substitution challenging. Furthermore, a large number of taxa and metabolites are yet undiscovered, though efforts at generating databases of combined microbiome-metabolome datasets are attempting to close this gap in knowledge [29]. In the following sections we will focus on selected findings in IBD patients.

## Disrupted metabolic and microbial signatures in IBD

Evidence for the importance of the microbiota in the pathophysiology of IBD has been accumulating for several decades [30]. Patients with CD and UC exhibit signs of dysbiosis, meaning that their microbiota is generally altered compared to healthy controls. Although the interindividual composition of the microbiota can differ largely between patients, one consistent finding confirmed by a recent meta-analysis is the decreased alpha-diversity in patients with CD and UC [31, 32]. This can have relevant implications, as a lower diversity in the gut likely leads to a decreased resilience and a higher susceptibility to external perturbations of the microbial community [33]. Numerous studies over the years have tried to identify patterns in the bacterial taxonomy of IBD patients compared to control patients. This has proven difficult, as many findings were not replicable across cohorts. On one hand, this is likely due to the high heterogeneity in the methods used to assess the bacteria in the different cohorts [34]. On the other hand, factors such as geographical location, diet and intrapersonal variability impact the microbiota [35] and make replication highly challenging across cohorts. *Erysipelatoclostridium* and *Tyzzerella 4* were among the bacterial genera consistently increased upon meta-analysis, while *Prevotella 9* and *Lachnospiraceae NK4A136* group were decreased in both CD and UC. However, many other findings from the individual cohorts used for the analysis, such as the increase in *Escherichia* in CD, were not consistently present [32]. This lack in consensus of taxonomic changes led to a shift in the analysis of the microbiota away from pure taxonomy towards their metabolic capabilities. One possibility is the use of metagenomics analyses, which provides more information about the functional potential of the microbial community [36]. Indeed, metagenomics analyses have identified significant differences in enzymatic abundances and profiles in the IBD microbiome compared to healthy controls. These shifts could be attributed to two different mechanisms: on the one hand, bacterial species with higher abundances in the IBD patients such as *E. coli* in CD led to an increased representation of their enzymes, with *E. coli* alone largely accounting for 220 differentially abundant enzymes. On the other hand, enzymes shared across species were overall differentially abundant in IBD patients or controls, thus indicating a shift in the metabolic capabilities of the community [37]. Interestingly, the bacterial composition observed in patients with CD or UC seemed to be distinct from other intestinal or inflammatory diseases: Ning et al. found an AUROC of 0.66 to 0.95 when applying a machine learning method for the detection of IBD based on metagenomics data, but these signatures were not seen in colorectal cancer or type 2 diabetes [12]. While metagenomics may provide more information than taxonomy alone, the abundance of bacterial species estimated using metagenomics data does not necessarily correlate with their transcriptional activity [38]. This means that taxonomic classification and functional potential alone is not sufficient to quantify the metabolic contribution of the microbiota. Therefore, measuring the products of the bacterial metabolism directly provides a more accurate measure of the activity of the microbiota and factors that influence that metabolism.

The metabolic profiles of patients with CD and UC exhibit distinct features compared to those of healthy controls in various anatomical compartments, as systematically reviewed by Gallagher et al. [39]. Though the metabolome is highly individual, stool metabolites that were consistently altered in CD, UC or both included bile acids, which can be metabolized by certain microbial species. Furthermore, short chain fatty acids (SCFA) have been found to be decreased in fecal metabolomics of patients with IBD, possibly due to a depletion of butyrate-producing microbiota [39]. Amino acids such as tryptophan and glutamine have been found to be increased in the stool of patients with CD and UC, and an increase in compounds belonging to the tricarboxylic acid cycle in stool indicates an altered microbial energy metabolism [12]. In blood metabolomics, the amino acids glutamine and tryptophan have been detected at lower levels in IBD compared to controls, while isoleucine was generally increased [39]. While differences in metabolic profiles could in part be due to medication use in IBD, Daniluk et al. showed that serum alterations in metabolic signatures were already present in newly diagnosed children with CD and UC prior to therapy [40]. Strikingly, Vila et al. demonstrated that the predictive strength of the microbiome on fecal metabolites was higher than that of lifestyle, diet and genetics [41].

Furthermore, certain microbial or metabolic signatures have been associated with therapy response to various medications in IBD patients. The composition of the bacterial community was shown to change in patients with UC who responded to four-week long therapy with corticosteroids, with an increase of beneficial taxa and higher predicted butyrate production compared to baseline. Though the bacterial community did not differ at baseline between the responders and non-responders, there were minor longitudinal changes in patients who did not respond to corticosteroid treatment, and their bacterial composition overall was distinct from the responders after four weeks [42]. Due to the multi-factorial influence that the host has on its microbiota, changes to the microbiome following successful therapeutic interventions need to be interpreted with care: one cannot assume that changing microbial profiles directly influences disease course, as it is also likely that the microbiota have adapted to the environmental changes caused directly by the drug, or indirectly through the physiological and behavioral changes that occur as a patient responds to a given therapy. In a similar vein, metabolic changes resulting from therapeutic success cannot be directly attributed to the action of the differential metabolites when drugs, behaviors and physiological changes alone can influence metabolism. Nevertheless, for biologicals such as anti-integrin therapy, a predictive signature of therapy responders could already be identified at baseline using taxonomy and microbial pathways for CD and UC [43]. This was also seen in CD and UC patients undergoing anti-TNF therapy, in which the predicted metabolic interchange of the bacterial community at baseline was higher in responders and non-responders [44]. Similar findings showing distinct features in the microbial community at baseline of CD and UC patients who respond to treatment could also be shown for anti-cytokine therapy. Here, they also demonstrated the added value of supplementary omics-layers such as metabolomics and proteomics to clinical or metagenomics data for the prediction of therapy-response at baseline [45].

Given the alterations of both microbial and metabolic signatures in IBD, exploring the interplay between those two systems may enable us to deduct novel strategies for diagnostics, targeted therapies and disease monitoring. In the following sections, we describe compound classes that can be altered through microbial metabolism, their potential associations with IBD pathophysiology, and their potential exploitation for clinical utilization. Above that, we will address current challenges in the translation of novel scientific findings into everyday clinical practice.

## Short chain fatty acids

Short chain fatty acids (SCFA) are produced from microbial fermentation of dietary fiber. This class of organic acids are defined by their length, having less than six carbon atoms [46]. Although human cells can produce SCFA, microbial contribution is relatively high, as evidenced by studies where germ-free mice exhibited a 99% reduction in cecal SCFA relative to conventionally raised mice [47]. Within the class of SCFA, butyrate, propionate and acetate are among the most widely studied and have been demonstrated to modulate pro-inflammatory cytokine profiles *in-vitro*; however, the physiological function varies by individual SCFA entity [48]. Despite heterogeneity in the literature, a meta-analysis on SCFA levels found low fecal levels of acetate, propionate, butyrate, and valerate when comparing patients with IBD to healthy controls [49]. Of note, a study by Lloyd-Price and colleagues found changes to SCFA in IBD to be particularly related to periods of dysbiosis. In this case, dysbiosis was defined by a large deviation from the taxonomic composition of the non-IBD samples: as this was a longitudinal study, dysbiotic periods could be identified longitudinally within individual IBD patients [50]. This could explain the heterogeneity of the literature, as fluctuations between dysbiotic and non-dysbiotic periods likely result in increased variation in SCFA and this may affect statistical power. Furthermore, in longitudinal analyses of patients with CD and UC treated with an anti-TNF-α antibody, increased microbial production capacity of SCFA was associated with a better therapeutic outcome [44]. A similar association between increased microbial production capacity of SCFA and response to therapy has also been observed for other therapeutic principles (azathioprine, corticosteroids) [42, 51].

Considerable interest is directed towards butyrate, as it is reported to modulate intestinal motility, improve mucosal barrier integrity, and provide energy to colonocytes. In fact, the hypothesis that insufficient butyrate availability disrupts energy homeostasis in colonocytes, thereby driving UC-related inflammation was put forward in 1980 [52]. Early clinical trials involving butyrate enemas in UC yielded mixed results [53, 54]; potentially due to the compound's strong stench affecting patient compliance and, consequently, efficacy [55].

More recently, butyrate has been incorporated as a treatment in several clinical trials (Table 1). For example, 12-week oral supplementation of butyrate failed to improve disease symptoms in 29 newly diagnosed pediatric IBD patients relative to the 43-patient placebo group [56]. There is, however, an ongoing clinical trial (NCT05218850) aimed at assessing the efficacy of butyrate enemas in pediatric UC. In another interventional trial, oral butyrate supplementation resulted in quality of life (QoL) improvements of adult UC patients in the treatment arm as well as increases in the butyrate producing bacteria *Butyricicoccus* in CD and *Lachnospiraceae* in UC [57]. Strikingly, this is not the only study to report improvements to QoL upon butyrate supplementation in UC patients: supplementation with orally administered butyrate and medium-chain triglycerides improved sleep, QoL and additionally biochemical measures of inflammation (CRP and calprotectin) [58]. So far, there is no consensus on the optimal mode of delivery or dose for butyrate supplementation and the considerable variation in trial results is therefore not surprising. However, taken together, there is cause for consideration that butyrate could be an effective adjunct therapy for IBD management.

**Table 1:** Clinical trials involving direct administration of microbial metabolites to individuals with IBD. Clinicaltrials.gov was searched for trials involving "inflammatory bowel disease" and the listed treatment agents. Trials where the treatment agent was used in conjunction with another therapy have not been included. Successful search terms were butyrate, bile acids, tauroursoursodeoxycholic acid/TUDCA, nicotinamide and nicotinic. The following search terms did not produce additional relevant results: propionate, acetate, valerate, short chain fatty acids, SCFA, deoxycholic acid/DCA, ursodeoxycholic acid/UDCA, glycocholic acid/GCA, lithocholic acid/LCA, muricholic acid, taurocholic/TCA, taurolithocholic/TLCA, taurochenodeoxycholic/TCDCA, taurodeoxycholic/TDCA, indole, indole lactate/indole-3-lactate, indole-3-carbonyl, indole-3-acetic acid, acetic acid, tryptophan, kynurenine, kynurenate/kynurenic, xanthurenate/xanthurenic, serotonin, tryptamine, indole-3-ethanol, indole-3-pyruvate, AhR/aryl hydrocarbon, indole-3-aldehyde. Abbreviations: y.o.: years old. UCDA: ursodeoxycholic acid, TUDCA: tauroursoursodeoxycholic acid. NMN: Nicotinamide mononucleotide. NR: Nicotinamide riboside. CICR-NAM: Controlled-Ileocolonic-Release Nicotinamide. * Dietary intervention trial, placebo controlled

Reference ID	Treatment agent	Intervention details	Trial design	Target group	Study size	Trial status or outcome
NCT05456763	Butyrate	250 mg orally (2x daily, 12-weeks)	Randomized placebo-controlled	Pediatric IBD (6–18 y.o.)	29 (treatment), 43 (placebo)	No difference in disease activity
NCT05218850	Butyrate	Butyrate enema (1x daily, 12-weeks)	Phase I open label	Pediatric IBD (7–21 y.o.)	10 (estimated)	In recruitment
NCT04879914	Butyrate	600 mg total orally (60-days)	Randomized placebo-controlled	IBD (18–75 y.o.)	28 (treatment), 29 (placebo)	No difference in disease activity
NCT03724175	UDCA	300 mg orally (2x daily, 10-weeks)	Phase II/III open label	UC-related pouchitis (>18 y.o)	15 (estimated)	In recruitment
NCT04114292	TUDCA	1.75-2g total TUDCA daily (6-weeks)	Phase I open label	UC (18–65 y.o.)	13 (estimated)	Status unknown
NCT05258474	CICR-NAM	Single and multiple ascending doses (1, 2, 4 g orally)	Phase I double-blind	Healthy and IBD (18–75 y.o.)	49	Completed
NCT06488625	CICR-NAM	2 or 3 g per day orally for 52-weeks	Phase II/III double-blind	UC (18–80 y.o.)	459 (estimated)	Not yet recruiting
NCT06214078	NMN	250 mg NMN orally (2x daily, 8-weeks)	N/A*	UC (18–75 y.o.)	48 (estimated)	In recruitment
NCT05561738	NR	12.5mg/kg/day NR orally by participant weight.	N/A*	Pediatric UC (less than 18 y.o.)	40 (estimated)	In recruitment

## Bile acids

Primary bile acids (BA) are cholesterol derivatives that are produced by pericentral hepatocytes before being secreted into the small intestine, where they enhance absorption of nutrients including lipids, sterols and vitamins [59, 60]. While the vast majority of BA are recycled, a small proportion will enter the large intestine, where microbial transformation of primary BA into secondary BA takes place [59]. Finally, transformation of secondary BA by the liver results in tertiary BA [61]. Secondary BA exhibit a high level of diversity and have multiple effects on host cells. Their general mode of action is thought to be related to their hydrophobicity, which can lead to direct cell membrane damage, as well as via signaling cascades initiated by binding to membranes and nuclear receptors. The resulting physiological implication can be of pro- or anti-inflammatory nature [61]. While total BA concentrations in the stool are similar between individuals with IBD and the healthy population, the BA profile is altered in IBD and is characterized by a decrease in secondary BA. This effect is exacerbated during disease flares [62]. Furthermore, secondary BA in blood have been found to be significantly increased at baseline in patients achieving remission after 14 weeks of anti-cytokine therapy. This was likely due to the increased presence of microbial enzymes capable of converting primary to secondary BA through bile acid 7α/β-dehydroxylation prior to treatment in the responders [45]. Secondary BA might furthermore be considered as compounds with therapeutic potential: To date, at least two registered clinical trials exist involving secondary BA interventions, but no results can be reported yet (Table 1). An in-depth overview of the potential role of bile acids in IBD pathogenesis can be found in the works of Thomas et al. (2022) and Kumar et al. (2022) [59, 60].

## Polyamines

Polyamines are unique compounds in that they are hydrocarbons with an amino group at each end. As they are positively charged at physiological pH, they can bind to DNA and other negatively charged molecules. They are, as a result, highly biologically active and can exert a multiplicity of physiological effects [63]. For example, polyamines possess both pro- and anti-apoptotic properties and can affect the state of diverse immune cells (e.g., T cell differentiation and macrophage polarization) [64]. Along with humans, bacteria have the capacity to produce several polyamines using arginine as a precursor [65]. Injection of isotope-labelled arginine into rats via a colonic catheter resulted in accumulation of isotope-labelled putrescine, suggesting that microbiota can produce putrescine in-vivo, thereby modifying intestinal concentrations [66]. In stool, increased levels of the cytotoxins cadaverine and putrescine [67] have been identified in individuals with UC and CD relative to healthy controls [68, 69]. Examination of intestinal biopsies has revealed increased intracellular levels of spermidine and N^8^-acetylspermidine and decreased spermine in the colonic epithelial cells of individuals with IBD relative to healthy individuals [70]. Furthermore, spermidine, N^8^-acetylspermidine, and N^1^-acetylspermine all increased with inflammatory activity. The findings on spermidine are surprising given its commonly recognized health-promoting properties in aging research. One of its key benefits, particularly relevant to the pathophysiology of IBD, is the induction of autophagy [71]. Clearly, more research is warranted to exactly delineate the host-microbe co-metabolism of polyamines in the gut and their potential role in IBD pathophysiology.

## Tryptophan and derivatives, NAD^+^ metabolism

While amino acids can be used for protein synthesis, these chemical compounds can also be broken down into diverse bioactive derivatives. One classic example is the essential amino acid tryptophan (Trp), which can be catalyzed along three main biological routes: the kynurenine pathway, the serotonin pathway, and into various indolic compounds [72]. Serum levels of Trp are reduced in CD and UC, and there is a negative relationship between disease activity and serum Trp [73]. Many Trp catabolites act as agonists for the transcription factor aryl hydrocarbon receptor (AhR). Importantly, the result of AhR signaling depends not only on the interacting ligand, but also cell type and microenvironment [74]. However, it overall tends to favor anti-inflammatory responses, e.g. when Trp derivatives interact with the AhR to regulate gut barrier function [75]. Germ-free studies have implicated microbiota in mediating host levels of Trp and its derivatives and suggest that these changes are far-reaching: metabolic changes have been observed across organs and serum in germ-free animals relative to their conventionally raised or recolonized counterparts [76]. Moreover, several studies have demonstrated that administration of specific Trp derivatives (e.g., indole-3-pyruvic acid, indole-3-acetic acid [77, 78]) can reduce symptoms of experimentally induced colitis in mice. A recent study demonstrated the importance of considering the interaction between diet and microbial community ecology: levels of the generally pro-inflammatory compound indole can be reduced via metabolic interactions resulting from fiber fermentation by *Bacteroides thetaiotaomicron.* By cross-feeding monosaccharides to *E. coli*, production of indole by *E. coli* can be reduced, thereby providing additional Trp for *Clostridium sporogenes* to act as a substrate for indole-3-pyruvic acid and indole-3-lactic acid biosynthesis [46]. This study underlines the complexity of microbial metabolic contributions to their host and suggests that metabolic capacities of single microbial species should not be considered in isolation, but within the context of the microbial community, host behavior and physiology. Despite mounting evidence for a beneficial effect of Trp derivatives (especially AhR agonists), our search for interventions involving these compounds yielded no relevant results.

Importantly, degradation of Trp along the kynurenine pathway feeds one arm of nicotinamide adenine dinucleotide (NAD^+^) synthesis via the *de-novo* pathway. NAD^+^ is furthermore synthesized from nicotinamide (NAM), nicotinamide mononucleotide (NMN) and nicotinamide riboside (NR) via the salvage pathway and nicotinic acid (NA) via the Preiss-Handler-pathway [79–83]. Given NAD^+^ is an essential redox coenzyme, its deficiency has been associated with several conditions, including UC, and mitochondrial dysfunction [52, 84]. Of note, intestinal microbiota influence host NAD^+^ bioavailability: For example, in germ-free mice, a total reduction of NADH/NAD^+^ redox ratio in colonocytes has been observed [85]; and microbiota fulfil a critical function in supplying the host with NAD^+^ precursors, e.g. by converting NA into NAM [86, 87]. Although it has been discussed controversially whether high abundances of NAD^+^ are generally favorable in intestinal inflammation [88], increasing evidence suggests that supplementation with NAD^+^ precursors exerts beneficial effects on colitis severity in preclinical models [89]. Accordingly, treatment of moderately active UC patients with NA-containing enemas induced clinical remission [90]; while ileocolonic release of NAM has been assessed for its efficacy in healthy controls and IBD patients, with phase II/III clinical trials in UC patients set to begin recruitment soon, and recruitment for studies testing NMN and NR in UC are ongoing (Table 1).

## Modulation of microbial metabolism via microbiota alteration

Aside from direct application of microbial metabolites, it is possible to modulate microbial metabolism by influencing the overall microbial community. This can be in form of single-strain interventions, or by influencing microbial community structure. One reported single-strain utilized the probiotic strain *E. coli* Nissle 1917, which was found to be as effective as treatment with aminosalicylates in maintaining remission in UC [91]. Community changes can be achieved by several methods, e.g., oral supplementation of mixed-strain probiotics or prebiotics, or through fecal microbiota transplantation (FMT). In fact, a symbiotic combination of prebiotics and probiotics was able to reduce colitis activity in UC patients [92]. Furthermore, dietary fiber constitutes a relevant prebiotic as it can act as a substrate for SCFA production and its consumption reduces the risk of developing CD [93]. Of specific relevance for this review, increased production of anti-inflammatory secondary BA and SCFA are observed in UC patients who benefit from FMT [94].

To date, however, only few bacteria-based concepts have been transferred into clinical practice: this is majorly due to the challenging task of determining the microbial and metabolic signatures of an individual patient to allow for a tailored treatment approach targeting the pathophysiologically relevant “defects”. One example for this is a recent study by Armstrong et al., which suggests that fiber supplementation in patients who lack the required fiber fermenting microbiota might drive inflammation rather than alleviate it [95]. Additionally, their efficacy is often limited by low concentrations at the site of action, functional impairment caused by gastric acids and BA as well as inefficient colonization of beneficial microbial species. Transferring complex microbial communities also involves risks such as potential containment of pathobionts or unpredictable impacts on the host microbiota; therefore, it might be considered to transfer isolated strains of interest [96]. Despite these difficulties, several Phase I and II clinical trials have been initiated, and some have produced promising results. However, it remains to be seen whether the microbial therapeutics from these trials will be adopted into clinical practice. For a recent comprehensive overview of these trials, refer to the review by Bethlehem et al. [97].

## Barriers to clinical implementation

Despite the emerging potential to use microbial metabolic characteristics for diagnostic or therapeutic strategies in IBD, clinical implementations are rare. One limiting factor is the descriptive nature of microbiota analyses, especially when using 16S rRNA gene sequencing as the only tool. Although we are just beginning to explore species and strain-level genomic variation and their associated consequences for metabolic plasticity, clearly this method is limited in its ability to provide a representation of the respective metabolic potential. It is therefore inadequate for use as a diagnostic tool in this setting although its utility is widely propagated by commercial entities and on social media. Moreover, inter-individual variation in microbial and metabolic signatures are quite high both in the healthy population and within the population of individuals with IBD. This reduces the predictive and diagnostic power of single-omics layers and often renders *one-fits-all*-approaches ineffective. The high interindividual variability can be addressed through careful recruitment practices that ensure a relatively homogenous population, or alternatively, through recruitment of large cohorts where clustering approaches can be applied to identify distinct subpopulations. For a global assessment of the complex actors and their interplay in an organism, an integration of multi-omics-based data sets has been suggested [98]. A joint analysis of metabolomics and 16S sequencing/metagenomics data is an obvious starting point to acquire a multidimensional, functional classification of microbiota-host interaction. Han et al. for example describe a method that uses a metabolite library to infer which metabolic signatures are produced by which microbes [99], and a recent study in *Microbiome* introduces LOCATE, a machine learning tool to predict metabolite concentrations, microbiome-metabolome relationships and host conditions [100]. Combining -omics layers can aid in the identification of high-quality features, as cross-correlated features are more likely to convey biologically or clinically meaningful information. Ultimately, these carefully selected features can be used to design more cost-effective screening tools for larger, prospective clinical trials or even in-vitro screening. To account for low reproducibility due to highly diverse study designs of IBD trials, a recent cross-cohort integrative analysis has identified and validated several microbial species, genes and metabolites to characterize IBD and distinguish it from other disease entities [12]. Along the same line, Muller et al. make an online collection of cross-study paired microbiome-metabolome datasets publicly available for broad functional characterization [29]. Such approaches, which are based on deriving general principles by integrating multiple individual microbiome-related metabolic fingerprints, have great revolutionary potential: after functional characterization of the microbial composition and the pathophysiologically relevant metabolites, the corresponding signaling pathways can be modified following a targeted approach with the help of pre-, pro- and postbiotics.

## Outlook

As technology advances, combining -omics layers has become easier and more cost-effective. These changes are facilitating a new understanding of the repercussions of host-microbe interactions on human physiology, which will leverage the creation of new diagnostic and monitoring tools in the IBD field over the coming years. There are also several clinical trials that either directly supplement microbial metabolites (e.g., butyrate) or aim to modulate host-microbe interactions through influencing microbial community structure. High inter-individual variation and incompletely investigated interplay between host and microbiota does still present an impediment to progress in this area. This may be circumvented by homogenizing patient recruitment (e.g., separate analysis of CD and UC) and standardizing protocols for clinical trials and through application of tailored interventions based on individual patient-omics profiles. Furthermore, owing to the nature of the diseases, the nutritional environment of the microbiota is expected to differ significantly between individuals with and without IBD (e.g., due to impairment in nutritional uptake or dietary changes). This alone is likely enough to influence microbial metabolism in the inflamed gut and therefore changes to microbial communities or metabolites do not necessarily necessitate inflammatory consequences. This problem can be partially overcome through validation across culturally diverse populations which will dilute diet-driven signals. It remains an open question as to whether there are distinct subpopulations of patients who would differentially respond to therapeutic interventions. We believe that using clustering approaches to identify these groups (e.g., clustering of metabolomics data to identify discrete “metabotypes”) represents a promising way forward to address the heterogeneity of the patient population and one that could pave the way towards prospective studies which are sorely needed on the path towards clinical implementation. As we hope can be appreciated from Table 1, several prospective clinical trials are ongoing that will leverage the existing literature to assess the influence of microbial metabolites on disease activity in IBD. This, too, represents an important approach for clinical translation of the wealth of findings that have been produced thus far, and there are still more microbial metabolites which may be well-suited for this study design, including other SCFA beyond butyrate and tryptophan metabolites.

In summary, targeted manipulation of microbiota and microbial metabolites in IBD remains underutilized in clinical practice. Harnessing the advances in multi-omics research to craft personalized targeted intervention strategies and diagnostic markers could unlock the potential of microbial metabolism for disease modulation, setting the stage for its adoption in precision medicine.

## Key statements

While 16S sequencing has advanced our understanding of the composition of the microbiota, employed on its own it does not generate an in-depth understanding of IBD pathophysiology, as it insufficiently provides insight into the pathophysiologically highly relevant functional capabilities of microbial strains

The limited success of pre-, pro- and postbiotics in treating IBD highlights the complexity of the interplay of different actors that needs to be addressed in order to target the critical pathologic components driving inflammation. These endeavors are compounded by large variability across IBD patients

Applying integrated multiomics-based approaches can generate a holistic picture of the complex interaction between host and microbe, helping to identify microbial and metabolic signatures and imbalances associated with IBD as a foundation for personalized diagnostics and targeted therapies

To conduct effective precision medicine, study designs, analytical techniques and clinical practices need to be standardized. Personalized diagnostic and treatment regimens based on individual microbiota and metabolome profiles bear a high potential to improve outcomes in IBD management

## Data Availability

There are no primary data used for analysis. Conclusions were obtained based on literature review.

## References

[1] Sender R, Fuchs S, Milo R (2016) Are We Really Vastly Outnumbered? Revisiting the Ratio of Bacterial to Host Cells in Humans. Cell. 164(3):337–40 26824647 10.1016/j.cell.2016.01.013

[2] Nash AK, Auchtung TA, Wong MC, Smith DP, Gesell JR et al (2017) The gut mycobiome of the Human Microbiome Project healthy cohort. Microbiome. 5:153 29178920 10.1186/s40168-017-0373-4 PMC5702186

[3] Shkoporov AN, Clooney AG, Sutton TDS, Ryan FJ, Daly KM et al (2019) The Human Gut Virome Is Highly Diverse, Stable, and Individual Specific. Cell Host Microbe. 26(4):527-541.e5 31600503 10.1016/j.chom.2019.09.009

[4] Chibani CM, Mahnert A, Borrel G, Almeida A, Werner A et al (2022) A catalogue of 1,167 genomes from the human gut archaeome. Nat. Microbiol. 7(1):48–61 34969981 10.1038/s41564-021-01020-9 PMC8727293

[5] Pollard M, Sharon N (1970) Responses of the Peyer’s Patches in Germ-Free Mice to Antigenic Stimulation. Infect Immun 2(1):96–100 16557807 10.1128/iai.2.1.96-100.1970 PMC415970

[6] Ivanov II, de Frutos RL, Manel N, Yoshinaga K, Rifkin DB et al (2008) Specific Microbiota Direct the Differentiation of IL-17-Producing T-Helper Cells in the Mucosa of the Small Intestine. Cell Host Microbe 4(4):337–49 18854238 10.1016/j.chom.2008.09.009 PMC2597589

[7] Smith PM, Howitt MR, Panikov N, Michaud M, Gallini CA et al (2013) The microbial metabolites, short-chain fatty acids regulate colonic treg cell homeostasis. Science 341(6145):569–73 23828891 10.1126/science.1241165 PMC3807819

[8] Arpaia N, Campbell C, Fan X, Dikiy S, van der Veeken J et al (2013) Metabolites produced by commensal bacteria promote peripheral regulatory T cell generation. Nature 504(7480):451–55 24226773 10.1038/nature12726 PMC3869884

[9] Furusawa Y, Obata Y, Fukuda S, Endo TA, Nakato G et al (2013) Commensal microbe-derived butyrate induces the differentiation of colonic regulatory T cells. Nature 504(7480):446–50 24226770 10.1038/nature12721

[10] Fromentin S, Forslund SK, Chechi K, Aron-Wisnewsky J, Chakaroun R et al (2022) Microbiome and metabolome features of the cardiometabolic disease spectrum. Nat. Med. 28(2):303–14 35177860 10.1038/s41591-022-01688-4 PMC8863577

[11] Yu D, Du J, Pu X, Zheng L, Chen S et al (2022) The Gut Microbiome and Metabolites Are Altered and Interrelated in Patients With Rheumatoid Arthritis. Front. Cell. Infect. Microbiol. 11:763507 35145919 10.3389/fcimb.2021.763507 PMC8821809

[12] Ning L, Zhou Y-L, Sun H, Zhang Y, Shen C et al (2023) Microbiome and metabolome features in inflammatory bowel disease via multi-omics integration analyses across cohorts. Nat. Commun. 14(1):7135 37932270 10.1038/s41467-023-42788-0 PMC10628233

[13] Tan H, Shi Y, Yue T, Zheng D, Luo S, et al. 2023. Machine learning approach reveals microbiome, metabolome, and lipidome profiles in type 1 diabetes. J Adv Res 72(Supplement_1) 10.1016/j.jare.2023.11.025 PMC11464464 38042287

[14] Brown EM, Ke X, Hitchcock D, Jeanfavre S, Avila-Pacheco J et al (2019) *Bacteroides*-Derived Sphingolipids Are Critical for Maintaining Intestinal Homeostasis and Symbiosis. Cell Host Microbe 25(5):668-680.e7 31071294 10.1016/j.chom.2019.04.002 PMC6544385

[15] Paik D, Yao L, Zhang Y, Bae S, D’Agostino GD et al (2022) Human gut bacteria produce ΤΗ17-modulating bile acid metabolites. Nature 603(7903):907–12 35296854 10.1038/s41586-022-04480-z PMC9132548

[16] Sun H, Guo Y, Wang H, Yin A, Hu J et al (2023) Gut commensal Parabacteroides distasonis alleviates inflammatory arthritis. Gut 72(9):1664–77 36604114 10.1136/gutjnl-2022-327756

[17] de Souza HSP, Fiocchi C, Iliopoulos D (2017) The IBD interactome: an integrated view of aetiology, pathogenesis and therapy. Nat Rev Gastroenterol Hepatol 14(12):739–49 28831186 10.1038/nrgastro.2017.110

[18] Alatab S, Sepanlou SG, Ikuta K, Vahedi H, Bisignano C et al (2020) The global, regional, and national burden of inflammatory bowel disease in 195 countries and territories, 1990–2017: a systematic analysis for the Global Burden of Disease Study 2017. Lancet Gastroenterol Hepatol 5(1):17–30 31648971 10.1016/S2468-1253(19)30333-4 PMC7026709

[19] Singh S, Murad MH, Fumery M, Sedano R, Jairath V et al (2021) Comparative efficacy and safety of biologic therapies for moderate-to-severe Crohn’s disease: a systematic review and network meta-analysis. Lancet Gastroenterol Hepatol 6(12):1002–14 34688373 10.1016/S2468-1253(21)00312-5 PMC8933137

[20] Khan I, Ullah N, Zha L, Bai Y, Khan A et al (2019) Alteration of Gut Microbiota in Inflammatory Bowel Disease (IBD): Cause or Consequence? IBD Treatment Targeting the Gut Microbiome. Pathogens 8(3):126 31412603 10.3390/pathogens8030126 PMC6789542

[21] Lavelle A, Sokol H (2020) Gut microbiota-derived metabolites as key actors in inflammatory bowel disease. Nat Rev Gastroenterol Hepatol 17(4):223–37 32076145 10.1038/s41575-019-0258-z

[22] Rühlemann MC, Hermes BM, Bang C, Doms S, Moitinho-Silva L et al (2021) Genome-wide association study in 8,956 German individuals identifies influence of ABO histo-blood groups on gut microbiome. Nat Genet 53(2):147–55 33462482 10.1038/s41588-020-00747-1

[23] Zhernakova DV, Wang D, Liu L, Andreu-Sánchez S, Zhang Y et al (2024) Host genetic regulation of human gut microbial structural variation. Nature 625(7996):813–21 38172637 10.1038/s41586-023-06893-w PMC10808065

[24] Rinninella E, Raoul P, Cintoni M, Franceschi F, Miggiano GAD et al (2019) What is the Healthy Gut Microbiota Composition? A Changing Ecosystem across Age, Environment, Diet, and Diseases. Microorganisms 7(1):14 30634578 10.3390/microorganisms7010014 PMC6351938

[25] Zheng D, Liwinski T, Elinav E (2020) Interaction between microbiota and immunity in health and disease. Cell Res 30(6):492–506 32433595 10.1038/s41422-020-0332-7 PMC7264227

[26] Postler TS, Ghosh S (2017) Understanding the Holobiont: How microbial metabolites affect human health and shape the immune system. Cell Metab 26(1):110–30 28625867 10.1016/j.cmet.2017.05.008 PMC5535818

[27] Ye S, Shah BR, Li J, Liang H, Zhan F et al (2022) A critical review on interplay between dietary fibers and gut microbiota. Trends Food Sci Technol 124:237–49

[28] Neveu V, Nicolas G, Amara A, Salek RM, Scalbert A (2023) The human microbial exposome: expanding the Exposome-Explorer database with gut microbial metabolites. Sci Rep 13(1):1946 36732606 10.1038/s41598-022-26366-w PMC9894932

[29] Muller E, Algavi YM, Borenstein E (2022) The gut microbiome-metabolome dataset collection: a curated resource for integrative meta-analysis. Npj Biofilms Microbiomes 8(1):1–7 36243731 10.1038/s41522-022-00345-5 PMC9569371

[30] Podolsky DK (2002) Inflammatory Bowel Disease. N Engl J Med 347(6):417–29 12167685 10.1056/NEJMra020831

[31] Ott SJ, Musfeldt M, Wenderoth DF, Hampe J, Brant O et al (2004) Reduction in diversity of the colonic mucosa associated bacterial microflora in patients with active inflammatory bowel disease. Gut 53(5):685–93 15082587 10.1136/gut.2003.025403 PMC1774050

[32] Abdel-Rahman LIH, Morgan XC (2023) Searching for a Consensus Among Inflammatory Bowel Disease Studies: A Systematic Meta-Analysis. Inflamm Bowel Dis 29(1):125–39 36112501 10.1093/ibd/izac194 PMC9825291

[33] Sommer F, Anderson JM, Bharti R, Raes J, Rosenstiel P (2017) The resilience of the intestinal microbiota influences health and disease. Nat Rev Microbiol 15(10):630–38 28626231 10.1038/nrmicro.2017.58

[34] Pittayanon R, Lau JT, Leontiadis GI, Tse F, Yuan Y et al (2020) Differences in Gut Microbiota in Patients With vs Without Inflammatory Bowel Diseases: A Systematic Review. Gastroenterology 158(4):930-946.e1 31812509 10.1053/j.gastro.2019.11.294

[35] Clooney AG, Eckenberger J, Laserna-Mendieta E, Sexton KA, Bernstein MT et al (2021) Ranking microbiome variance in inflammatory bowel disease: a large longitudinal intercontinental study. Gut 70(3):499–510 32536605 10.1136/gutjnl-2020-321106 PMC7873428

[36] Franzosa EA, Hsu T, Sirota-Madi A, Shafquat A, Abu-Ali G et al (2015) Sequencing and beyond: integrating molecular “omics” for microbial community profiling. Nat Rev Microbiol 13(6):360–72 25915636 10.1038/nrmicro3451 PMC4800835

[37] Franzosa EA, Sirota-Madi A, Avila-Pacheco J, Fornelos N, Haiser HJ et al (2019) Gut microbiome structure and metabolic activity in inflammatory bowel disease. Nat Microbiol 4(2):293–305 30531976 10.1038/s41564-018-0306-4 PMC6342642

[38] Schirmer M, Franzosa EA, Lloyd-Price J, McIver LJ, Schwager R et al (2018) Dynamics of metatranscription in the inflammatory bowel disease gut microbiome. Nat Microbiol 3(3):337–46 29311644 10.1038/s41564-017-0089-z PMC6131705

[39] Gallagher K, Catesson A, Griffin JL, Holmes E, Williams HRT (2021) Metabolomic Analysis in Inflammatory Bowel Disease: A Systematic Review. J Crohns Colitis 15(5):813–26 33175138 10.1093/ecco-jcc/jjaa227

[40] Daniluk U, Daniluk J, Kucharski R, Kowalczyk T, Pietrowska K et al (2019) Untargeted Metabolomics and Inflammatory Markers Profiling in Children With Crohn’s Disease and Ulcerative Colitis—A Preliminary Study. Inflamm Bowel Dis 25(7):1120–28 30772902 10.1093/ibd/izy402

[41] Vila AV, Hu S, Andreu-Sánchez S, Collij V, Jansen BH et al (2023) Faecal metabolome and its determinants in inflammatory bowel disease. Gut 72(8):1472–85 36958817 10.1136/gutjnl-2022-328048 PMC10359577

[42] Blesl A, Wurm P, Waschina S, Gröchenig HP, Novacek G et al (2024) Prediction of Response to Systemic Corticosteroids in Active UC by Microbial Composition—A Prospective Multicenter Study. Inflamm Bowel Dis 30(1):9–19 37463118 10.1093/ibd/izad126 PMC10769779

[43] Ananthakrishnan AN, Luo C, Yajnik V, Khalili H, Garber JJ et al (2017) Gut Microbiome Function Predicts Response to Anti-integrin Biologic Therapy in Inflammatory Bowel Diseases. Cell Host Microbe 21(5):603-610.e3 28494241 10.1016/j.chom.2017.04.010 PMC5705050

[44] Aden K, Rehman A, Waschina S, Pan W-H, Walker A et al (2019) Metabolic Functions of Gut Microbes Associate With Efficacy of Tumor Necrosis Factor Antagonists in Patients With Inflammatory Bowel Diseases. Gastroenterology 157(5):1279-1292.e11 31326413 10.1053/j.gastro.2019.07.025

[45] Lee JWJ, Plichta D, Hogstrom L, Borren NZ, Lau H et al (2021) Multi-omics reveal microbial determinants impacting responses to biologic therapies in Inflammatory Bowel Disease. Cell Host Microbe 29(8):1294-1304.e4 34297922 10.1016/j.chom.2021.06.019 PMC8366279

[46] Sinha AK, Laursen MF, Brinck JE, Rybtke ML, Hjørne AP et al (2024) Dietary fibre directs microbial tryptophan metabolism via metabolic interactions in the gut microbiota. Nat Microbiol 9(8):1964–78 38918470 10.1038/s41564-024-01737-3 PMC11306097

[47] Høverstad T, Midtvedt T (1986) Short-Chain Fatty Acids in Germfree Mice and Rats. J Nutr 116(9):1772–76 3761032 10.1093/jn/116.9.1772

[48] Tedelind S, Westberg F, Kjerrulf M, Vidal A (2007) Anti-inflammatory properties of the short-chain fatty acids acetate and propionate: A study with relevance to inflammatory bowel disease. World J Gastroenterol WJG 13(20):2826–32 17569118 10.3748/wjg.v13.i20.2826 PMC4395634

[49] Zhuang X, Li T, Li M, Huang S, Qiu Y et al (2019) Systematic Review and Meta-analysis: Short-Chain Fatty Acid Characterization in Patients With Inflammatory Bowel Disease. Inflamm Bowel Dis 25(11):1751–63 31498864 10.1093/ibd/izz188

[50] Lloyd-Price J, Arze C, Ananthakrishnan AN, Schirmer M, Avila-Pacheco J et al (2019) Multi-omics of the gut microbial ecosystem in inflammatory bowel diseases. Nature 569(7758):655–62 31142855 10.1038/s41586-019-1237-9 PMC6650278

[51] Effenberger M, Reider S, Waschina S, Bronowski C, Enrich B et al (2021) Microbial Butyrate Synthesis Indicates Therapeutic Efficacy of Azathioprine in IBD Patients. J Crohns Colitis 15(1):88–98 32687146 10.1093/ecco-jcc/jjaa152

[52] Roediger WE (1980) The colonic epithelium in ulcerative colitis: an energy-deficiency disease? Lancet Lond Engl 2(8197):712–15 10.1016/s0140-6736(80)91934-0 6106826

[53] Scheppach W, Sommer H, Kirchner T, Paganelli G-M, Bartram P et al (1992) Effect of butyrate enemas on the colonic mucosa in distal ulcerative colitis. Gastroenterology 103(1):51–56 1612357 10.1016/0016-5085(92)91094-k

[54] Steinhart AH, Hiruki T, Brzezinski A, Baker JP (1996) Treatment of left-sided ulcerative colitis with butyrate enemas: a controlled trial. Aliment Pharmacol Ther 10(5):729–36 8899080 10.1046/j.1365-2036.1996.d01-509.x

[55] Aden K, Schreiber S, Rosenstiel P (2020) Reply. Gastroenterology 158(5):1512–13 32068024 10.1053/j.gastro.2020.02.021

[56] Pietrzak A, Banasiuk M, Szczepanik M, Borys-Iwanicka A, Pytrus T et al (2022) Sodium Butyrate Effectiveness in Children and Adolescents with Newly Diagnosed Inflammatory Bowel Diseases—Randomized Placebo-Controlled Multicenter Trial. Nutrients 14(16):3283 36014789 10.3390/nu14163283 PMC9414716

[57] Facchin S, Vitulo N, Calgaro M, Buda A, Romualdi C et al (2020) Microbiota changes induced by microencapsulated sodium butyrate in patients with inflammatory bowel disease. Neurogastroenterol Motil 32(10):e13914 32476236 10.1111/nmo.13914 PMC7583468

[58] Firoozi D, Masoumi SJ, Mohammad-Kazem Hosseini Asl S, Labbe A, Razeghian-Jahromi I et al (2024) Effects of short-chain fatty acid-butyrate supplementation on expression of circadian-clock genes, sleep quality, and inflammation in patients with active ulcerative colitis: a double-blind randomized controlled trial. Lipids Health Dis 23(1):216 39003477 10.1186/s12944-024-02203-z PMC11245831

[59] Kumar A, Al-Hassi HO, Steed H, Phipps O, Brookes MJ (2022) Bile Acids and the Microbiome: Making Sense of This Dynamic Relationship in Their Role and Management in Crohn’s Disease. Can J Gastroenterol Hepatol 2022(1):8416578 35360442 10.1155/2022/8416578 PMC8964223

[60] Thomas JP, Modos D, Rushbrook SM, Powell N, Korcsmaros T (2022) The Emerging Role of Bile Acids in the Pathogenesis of Inflammatory Bowel Disease. Front, Immunol, p 13 10.3389/fimmu.2022.829525 PMC8850271 35185922

[61] Ridlon JM, Gaskins HR (2024) Another renaissance for bile acid gastrointestinal microbiology. Nat Rev Gastroenterol Hepatol 21(5):348–64 38383804 10.1038/s41575-024-00896-2 PMC11558780

[62] Duboc H, Rajca S, Rainteau D, Benarous D, Maubert M-A et al (2013) Connecting dysbiosis, bile-acid dysmetabolism and gut inflammation in inflammatory bowel diseases. Gut 62(4):531–39 22993202 10.1136/gutjnl-2012-302578

[63] Kurihara S (2022) Polyamine metabolism and transport in gut microbes. Biosci Biotechnol Biochem 86(8):957–66 35648468 10.1093/bbb/zbac080

[64] Li JY, Guo YC, Zhou HF, Yue TT, Wang FX et al (2023) Arginine metabolism regulates the pathogenesis of inflammatory bowel disease. Nutr Rev 81(5):578–86 36040377 10.1093/nutrit/nuac070 PMC10086623

[65] Burrell M, Hanfrey CC, Murray EJ, Stanley-Wall NR, Michael AJ (2010) Evolution and multiplicity of arginine decarboxylases in polyamine biosynthesis and essential role in Bacillus subtilis biofilm formation. J Biol Chem 285(50):39224–38 20876533 10.1074/jbc.M110.163154 PMC2998088

[66] Nakamura A, Ooga T, Matsumoto M (2019) Intestinal luminal putrescine is produced by collective biosynthetic pathways of the commensal microbiome. Gut Microbes 10(2):159–71 30183487 10.1080/19490976.2018.1494466 PMC6546329

[67] del Rio B, Redruello B, Linares DM, Ladero V, Ruas-Madiedo P et al (2019) The biogenic amines putrescine and cadaverine show in vitro cytotoxicity at concentrations that can be found in foods. Sci Rep 9(1):120 30644398 10.1038/s41598-018-36239-w PMC6333923

[68] Liu M, Guo S, Wang L (2024) Systematic review of metabolomic alterations in ulcerative colitis: unveiling key metabolic signatures and pathways. Ther Adv Gastroenterol 17:17562848241239580 38560428 10.1177/17562848241239580 PMC10981261

[69] Santoru ML, Piras C, Murgia A, Palmas V, Camboni T et al (2017) Cross sectional evaluation of the gut-microbiome metabolome axis in an Italian cohort of IBD patients. Sci Rep 7(1):9523 28842640 10.1038/s41598-017-10034-5 PMC5573342

[70] Weiss TS, Herfarth H, Obermeier F, Ouart J, Vogl D et al (2004) Intracellular Polyamine Levels of Intestinal Epithelial Cells in Inflammatory Bowel Disease. Inflamm Bowel Dis 10(5):529–35 15472512 10.1097/00054725-200409000-00006

[71] Madeo F, Eisenberg T, Pietrocola F, Kroemer G (2018) Spermidine in health and disease. Science 359(6374):eaan2788 29371440 10.1126/science.aan2788

[72] Agus A, Planchais J, Sokol H (2018) Gut microbiota regulation of tryptophan metabolism in health and disease. Cell Host Microbe 23(6):716–24 29902437 10.1016/j.chom.2018.05.003

[73] Harris DMM, Szymczak S, Schuchardt S, Labrenz J, Tran F et al (2024) Tryptophan degradation as a systems phenomenon in inflammation – an analysis across 13 chronic inflammatory diseases. eBioMedicine 102:105056 38471395 10.1016/j.ebiom.2024.105056 PMC10943670

[74] Cannon AS, Nagarkatti PS, Nagarkatti M (2021) Targeting AhR as a Novel Therapeutic Modality against Inflammatory Diseases. Int J Mol Sci 23(1):288 35008717 10.3390/ijms23010288 PMC8745713

[75] Scott SA, Fu J, Chang PV (2020) Microbial tryptophan metabolites regulate gut barrier function via the aryl hydrocarbon receptor. Proc Natl Acad Sci 117(32):19376–87 32719140 10.1073/pnas.2000047117 PMC7431026

[76] Liu B, Yu D, Sun J, Wu X, Xin Z et al (2022) Characterizing the influence of gut microbiota on host tryptophan metabolism with germ-free pigs. Anim Nutr 11:190–200 36263410 10.1016/j.aninu.2022.07.005 PMC9562448

[77] Aoki R, Aoki-Yoshida A, Suzuki C, Takayama Y (2018) Indole-3-Pyruvic Acid, an Aryl Hydrocarbon Receptor Activator, Suppresses Experimental Colitis in Mice. J Immunol Baltim Md 1950 201(12):3683–93 10.4049/jimmunol.1701734 30429284

[78] Qu X, Song Y, Li Q, Xu Q, Li Y et al (2024) Indole-3-acetic acid ameliorates dextran sulfate sodium-induced colitis via the ERK signaling pathway. Arch Pharm Res 47(3):288–99 38489148 10.1007/s12272-024-01488-z

[79] Ikeda M, Tsuji H, Nakamura S, Ichiyama A, Nishizuka Y, Hayaishi O (1965) Studies on the biosynthesis of nicotinamide adenine dinucleotide. ii. a role of picolinic carboxylase in the biosynthesis of nicotinamide adenine dinucleotide from tryptophan in mammals. J Biol Chem 240:1395–1401 14284754

[80] Schweiger M, Hennig K, Lerner F, Niere M, Hirsch-Kauffmann M et al (2001) Characterization of recombinant human nicotinamide mononucleotide adenylyl transferase (NMNAT), a nuclear enzyme essential for NAD synthesis. FEBS Lett 492(1–2):95–100 11248244 10.1016/s0014-5793(01)02180-9

[81] Rongvaux A, Shea RJ, Mulks MH, Gigot D, Urbain J et al (2002) Pre-B-cell colony-enhancing factor, whose expression is up-regulated in activated lymphocytes, is a nicotinamide phosphoribosyltransferase, a cytosolic enzyme involved in NAD biosynthesis. Eur J Immunol 32(11):3225–34 12555668 10.1002/1521-4141(200211)32:11<3225::AID-IMMU3225>3.0.CO;2-L

[82] Revollo JR, Grimm AA, Imai S (2004) The NAD Biosynthesis Pathway Mediated by Nicotinamide Phosphoribosyltransferase Regulates Sir2 Activity in Mammalian Cells*. J Biol Chem 279(49):50754–63 15381699 10.1074/jbc.M408388200

[83] Bieganowski P, Brenner C (2004) Discoveries of nicotinamide riboside as a nutrient and conserved NRK genes establish a Preiss-Handler independent route to NAD+ in fungi and humans. Cell 117(4):495–502 15137942 10.1016/s0092-8674(04)00416-7

[84] Kang YH, Tucker SA, Quevedo SF, Inal A, Korzenik JR, Haigis MC (2022) Metabolic analyses reveal dysregulated NAD+ metabolism and altered mitochondrial state in ulcerative colitis. PloS One 17(8):e0273080 35976971 10.1371/journal.pone.0273080 PMC9385040

[85] Donohoe DR, Garge N, Zhang X, Sun W, O’Connell TM et al (2011) The microbiome and butyrate regulate energy metabolism and autophagy in the mammalian colon. Cell Metab 13(5):517–26 21531334 10.1016/j.cmet.2011.02.018 PMC3099420

[86] Chellappa K, McReynolds MR, Lu W, Zeng X, Makarov M et al (2022) NAD precursors cycle between host tissues and the gut microbiome. Cell Metab 34(12):1947-1959.e5 36476934 10.1016/j.cmet.2022.11.004 PMC9825113

[87] Shats I, Williams JG, Liu J, Makarov MV, Wu X et al (2020) Bacteria Boost Mammalian Host NAD Metabolism by Engaging the Deamidated Biosynthesis Pathway. Cell Metab 31(3):564-579.e7 32130883 10.1016/j.cmet.2020.02.001 PMC7194078

[88] Gerner RR, Klepsch V, Macheiner S, Arnhard K, Adolph TE et al (2018) NAD metabolism fuels human and mouse intestinal inflammation. Gut 67(10):1813–23 28877980 10.1136/gutjnl-2017-314241 PMC6145287

[89] Niño-Narvión J, Rojo-López MI, Martinez-Santos P, Rossell J, Ruiz-Alcaraz AJ et al (2023) NAD+ Precursors and Intestinal Inflammation: Therapeutic Insights Involving Gut Microbiota. Nutrients 15(13):2992 37447318 10.3390/nu15132992 PMC10346866

[90] Li J, Kong D, Wang Q, Wu W, Tang Y et al (2017) Niacin ameliorates ulcerative colitis via prostaglandin D2-mediated D prostanoid receptor 1 activation. EMBO Mol Med 9(5):571–88 28341703 10.15252/emmm.201606987 PMC5412792

[91] Losurdo G, Iannone A, Contaldo A, Ierardi E, Di Leo A, Principi M (2015) Escherichia coli Nissle 1917 in Ulcerative Colitis Treatment: Systematic Review and Meta-analysis. J Gastrointest Liver Dis JGLD 24(4):499–505 10.15403/jgld.2014.1121.244.ecn 26697577

[92] Furrie E, Macfarlane S, Kennedy A, Cummings JH, Walsh SV et al (2005) Synbiotic therapy (Bifidobacterium longum/Synergy 1) initiates resolution of inflammation in patients with active ulcerative colitis: a randomised controlled pilot trial. Gut 54(2):242–49 15647189 10.1136/gut.2004.044834 PMC1774839

[93] Ananthakrishnan AN, Khalili H, Konijeti GG, Higuchi LM, de Silva P et al (2013) A Prospective Study of Long-term Intake of Dietary Fiber and Risk of Crohn’s Disease and Ulcerative Colitis. Gastroenterology 145(5):970–77 23912083 10.1053/j.gastro.2013.07.050 PMC3805714

[94] Paramsothy S, Nielsen S, Kamm MA, Deshpande NP, Faith JJ et al (2019) Specific Bacteria and Metabolites Associated With Response to Fecal Microbiota Transplantation in Patients With Ulcerative Colitis. Gastroenterology 156(5):1440-1454.e2 30529583 10.1053/j.gastro.2018.12.001

[95] Armstrong HK, Bording-Jorgensen M, Santer DM, Zhang Z, Valcheva R et al (2023) Unfermented β-fructan Fibers Fuel Inflammation in Select Inflammatory Bowel Disease Patients. Gastroenterology 164(2):228–40 36183751 10.1053/j.gastro.2022.09.034

[96] Buffie CG, Bucci V, Stein RR, McKenney PT, Ling L et al (2015) Precision microbiome reconstitution restores bile acid mediated resistance to Clostridium difficile. Nature 517(7533):205–8 25337874 10.1038/nature13828 PMC4354891

[97] Bethlehem L, Estevinho MM, Grinspan A, Magro F, Faith JJ, Colombel J-F (2024) Microbiota therapeutics for inflammatory bowel disease: the way forward. Lancet Gastroenterol Hepatol 9(5):476–86 38604201 10.1016/S2468-1253(23)00441-7

[98] Schirmer M, Garner A, Vlamakis H, Xavier RJ (2019) Microbial genes and pathways in inflammatory bowel disease. Nat Rev Microbiol 17(8):497–511 31249397 10.1038/s41579-019-0213-6 PMC6759048

[99] Han S, Van Treuren W, Fischer CR, Merrill BD, DeFelice BC et al (2021) A metabolomics pipeline for mechanistic interrogation of the gut microbiome. Nature 595(7867):415–20 34262212 10.1038/s41586-021-03707-9 PMC8939302

[100] Shtossel O, Koren O, Shai I, Rinott E, Louzoun Y (2024) Gut microbiome-metabolome interactions predict host condition. Microbiome 12:24 38336867 10.1186/s40168-023-01737-1 PMC10858481

